# Grassmannians and Cluster Structures

**DOI:** 10.1007/s41980-021-00542-6

**Published:** 2021-04-22

**Authors:** Karin Baur

**Affiliations:** University of Graz (on leave) and University of Leeds CIMPA School, 4/2019, Isfahan, Iran

**Keywords:** Cluster algebras, Cluster categories, Grassmannians, Frieze patterns, Dimer models, Root systems, 13F60, 16G50, 82B20, 14M15

## Abstract

Cluster structures have been established on numerous algebraic varieties. These lectures focus on the Grassmannian variety and explain the cluster structures on it. The tools include dimer models on surfaces, associated algebras, and the study of associated module categories.

## Introduction

These notes are from a course taught at a CIMPA school in Isfahan in April 2019. The Grassmannian of *k*-subspaces in an *n*-dimensional space is a classical object in algebraic geometry. It has been studied a lot in recent years. This is partly due to the fact that its coordinate ring is a cluster algebra: In her work [32], Scott proved that the homogenous coordinate ring of the affine cone over the Grassmannian has a cluster algebra structure. She used certain Postnikov diagrams in the disk to exhibit clusters for this. The connection to the combinatorics of Postnikov diagrams has sparked a lot of interest. In their work [21] on partial flag varieties and preprojective algebras, the authors equip the coordinate ring of an affine open cell in the Grassmannian with the structure of a cluster category. Jensen, King, and Su introduced the Grassmannian cluster categories ${\mathcal {F}}_{k,n}$ in [25] (see Section 2.2), thus giving a categorification of Scott’s cluster algebra structure of the homogenous coordinate ring. They provide a bijection between reachable indecomposable objects of ${\mathcal {F}}_{k,n}$ and cluster variables in the associated cluster algebra. This correspondence has provided new understanding in both directions. In the finite types, both the cluster algebra and the cluster category are determined. However, in general, the categories ${\mathcal {F}}_{k,n}$ are of infinite type and so far not well understood. In these lectures, we recall the cluster algebras and cluster categories of the Grassmannian and some of the key ingredients for studying them.

## Grassmannians

In this section, we recall the main notions and tools needed to study them. We start with the definition of the Grassmannian, Sect. 1.1. Then, we explain how $\hbox {Gr}(2,n)$ can be equipped with the structure of a cluster algebra, Sect. 1.2. This is one of the first examples of a cluster algebra. Since it is related with triangulations of polygons, we then explain how to obtain a quiver (oriented graph) from a triangulation of a surface, Sect. 1.3. In the last two parts, Sects. 1.4 and 1.5, we recall Scott’s theorem on the cluster algebra structure for arbitrary Grassmannians and describe the quiver of a Postnikov diagram. A key reference for several parts is Section 9 in Marsh’s book on cluster algebras, [27].

### The Grassmannian as a Projective Variety

We first recall the exterior algebra and the definition of Plücker coordinates, which we can use to describe an embedding of the Grassmannian into projective space.

Let $V:={{\mathbb {C}}}^n$, the tensor algebra is defined as $T(V)={{\mathbb {C}}}\oplus V\oplus (V\otimes V) \oplus (V^{\otimes 3}) \oplus \dots$, and the **exterior algebra** is the quotient:

$$\begin{aligned} {\Lambda }(V)=T(V)/J, \end{aligned}$$

where *J* is the ideal of *T*(*V*) generated by $\{x\otimes x\mid x\in V\}$. We write the product in ${\Lambda }(V)$ as $(x,y)\mapsto x\wedge y$. The elements of ${\Lambda }(V)$ are called **alternating tensors**.

#### Lemma 1.1

For all $x,y\in V$, we have $x\wedge y= -y\wedge x$.

#### Exercise 1.2

Prove Lemma 1.1

The **kth exterior power** ${\Lambda }^k(V)$ is the subspace of ${\Lambda }(V)$ spanned by the products $v_1\wedge v_2\wedge \dots \wedge v_k$, $v_i\in V\ \forall \,i$:

$$\begin{aligned} {\Lambda }(V)=\bigoplus _{k=0}^{\infty }{\Lambda }^k(V) \end{aligned}$$

(this is a finite sum since $\dim V<\infty$). Let $e_1,\dots , e_n$ be the natural basis of *V*, $(e_i)_j=\delta _{ij}$. Then, the $e_{i_1}\wedge \dots \wedge e_{i_k}$ with $1\le i_1<\dots <i_k\le n$ form a basis of ${\Lambda }^k(V)$ (note that $\dim {\Lambda }^k(V)={n\atopwithdelims ()k}$).

For $x\in {\Lambda }^k(V)$, we write $p_{i_1,\dots , i_k}(x)$ for the coefficient of *x* in terms of this basis: $x=\sum _{i_1<\dots <i_k}p_{i_1,\dots , i_k}e_{i_1}\wedge \dots \wedge e_{i_k}$. In particular, the $p_{i_1,\dots , i_k}$ are linear maps ${\Lambda }^k(V)\rightarrow {{\mathbb {C}}}$.

An element $x\in {\Lambda }^k(V)$ is **decomposable** or **pure** if $x=v_1\wedge \dots \wedge v_k$ where $\{v_1,\dots , v_k\}$ is a linearly independent set of vectors in *V*.

#### Exercise 1.3

Check that the vectors $v_1,\dots , v_k$ are linearly dependent if and only if $v_1\wedge \dots \wedge v_k=0$.

Let $$v_1,\dots , v_k$$ be linearly independent vectors, $$k\le n$$. We can use them to form a $$k\times n$$ matrix $$M=(M_{ij})_{ij} $$ of rank *k* where $$M_{ij}=(v_i)_j$$, taking the $$v_i$$ as rows.

We use the following notation for the minor of *M* of rows $a_1,\dots , a_r$ and columns $b_1,\dots , b_r$ (for $1\le r\le k$):

$$\begin{aligned} \triangle _{b_1,\dots , b_r}^{a_1,\dots , a_r}(M). \end{aligned}$$

#### Lemma 1.4

Let $x=v_1\wedge \dots \wedge v_k\in {\Lambda }^k(V)$ be decomposable, *M* as above. Then, we have:

$$\begin{aligned} p_{i_1,\dots , i_k}(x)=\triangle _{i_1,\dots , i_k}^{1,2,\dots , k}(M). \end{aligned}$$

#### Exercise 1.5

Prove Lemma 1.4. Hint: use the expansion of the $v_i$ in terms of $e_1,\dots , e_n$.

We are now ready to describe the Grassmannian, by defining it first as a set and then as a projective variety. Let $1<k<n$. The **Grassmannian** $\hbox {Gr}(k,n)$ is the set of *k*-dimensional subspaces of $V={{\mathbb {C}}}^n$. Take $U\in \hbox {Gr}(k,n)$ and $\{v_1,\dots , v_k\}$ a basis of *U*. Consider:

$$\begin{aligned} w:=v_1\wedge \dots \wedge v_k\in {\Lambda }^k(V) \end{aligned}$$

(since the $v_i$s are linearly independent, $w\ne 0$, a decomposable alternating tensor).

Note that *w* does not depend on the choice of basis, up to multiplication by a non-zero scalar. If we associate with *w* all the coefficients $p_{i_1,\dots , i_k}(w)$, we get a well-defined element $(p_{i_1,\dots ,i_k}(w))_{i_1<\dots <i_k}$ of the projective space ${{\mathbb {P}}}^N$ for $N={n\atopwithdelims ()k}-1$. This gives us a map:

$$\begin{aligned} \varphi :\hbox {Gr}(k,n)\rightarrow {{\mathbb {P}}}^N. \end{aligned}$$

The $p_{i_1,\dots ,i_k}$ are called the **Plücker coordinates**. Note that in the definition of $\varphi$, we have chosen the indices to be strictly increasing.

We want to describe the image of $\varphi$. For this, we extend the definition of the $p_{i_1,\dots , i_k}$ to arbitrary (multi-) sets $\{i_1,\dots , i_k\}$ with $i_j\in [n]=\{1,\dots , n\}$ by setting $p_{i_1,\dots , i_k}=0$ if there are $r\ne s$, such that $i_r=i_s$ and by setting $p_{i_1,\dots , i_k}={{\,\mathrm{sgn}\,}}(\pi )p_{j_1,\dots ,j_k}$ in case the $i_1,\dots , i_k$ are distinct, $\{i_1,\dots , i_k\}=\{j_1,\dots , j_k\}$ with $1\le j_1<\dots <j_k\le n$ and $\pi$ is the permutation with $\pi (i_k)=j_k$ for all *k*. With this notation, we can describe the relations that the image of $\hbox {Gr}(k,n)$ under $\varphi$ will satisfy.

The **Plücker relations for** $\hbox {Gr}(k,n)$ are the relations:

1.1 $$\begin{aligned} \sum _{r=0}^k (-1)^r p_{i_1,\dots , i_{k-1},j_r} p_{j_0,\dots , \hat{j_r},\dots , j_k} \end{aligned}$$

where the sum is taken over all tuples $(i_1,\dots , i_{k-1})$, $(j_0,\dots , j_k)$ satisfying $1\le i_1<\dots <i_{k-1}\le n$ and $1\le j_0<\dots <j_k\le n$.

#### Exercise 1.6

Write the Plücker relations for $\hbox {Gr}(2,5)$.

#### Facts

1. $x\in {\Lambda }^k(V)$ is decomposable $\Longleftrightarrow$, the Plücker relations on *x* are 0;
2. The image $\hbox {im}\varphi \subseteq {{\mathbb {P}}}^N$ are the elements of ${{\mathbb {P}}}^N$ for which the Plücker relations are 0;
3. $\varphi :\hbox {Gr}(k,n)\rightarrow {{\mathbb {P}}}^N$ is injective. It is called the **Plücker embedding**;
4. $\hbox {im}\varphi$ is an irreducible projective variety, so $\hbox {Gr}(k,n)$ is an irreducible projective variety.

For the proofs: (2) follows from (1) and from the definition of $\varphi$. For (1)–(3): [24, Section 3.4] for (2),(3) [29, Section 14], for (4) W. Fulton, 1997, [19, Sections 8, and 9.]

#### Example 1.7

For $\hbox {Gr}(2,4)$, there is a single Plücker relation:

$$\begin{aligned} p_{12}p_{34} - p_{13}p_{24} + p_{14}p_{23}. \end{aligned}$$

#### Remark 1.8

Let $Y\subseteq {{\mathbb {P}}}^r$ be a projective variety, $R:={{\mathbb {C}}}[x_0,x_1,\dots , x_r]$ a graded ring, each $x_i$ of degree 1. The **homogenous ideal** *J* **(***Y***) of** *Y* is the ideal of *R* generated by the homogenous elements of *R* which vanish on *Y*. The **homogenous coordinate ring of** *Y* is ${{\mathbb {C}}}[Y]:=R/J(Y)$. There is a natural projection:

$$\begin{aligned} \mathrm{pr}: {{\mathbb {C}}}^{r+1}{\setminus } \{0\}\rightarrow {{\mathbb {P}}}^r,\quad (a_0,\dots , a_r)\mapsto [a_0:\ldots :a_r]. \end{aligned}$$

The **affine cone** *C* **(***Y***) over** *Y* is the preimage of *Y* under pr:

$$\begin{aligned} C(Y):=\mathrm{pr}^{-1}(Y)\cup \{0\} \end{aligned}$$

One can show that *C*(*Y*) is an affine variety whose ideal is *J*(*Y*) regarded as an ideal of *R* without the grading; the coordinate ring of *C*(*Y*) coincides with the homogenous coordinate ring of *Y*, [23, Exercise 2.10]. From the facts above, we get that the affine cone of $\hbox {Gr}(k,n)$ can be identified with the decomposable elements of ${\Lambda }^k(V)$ together with 0. And the coordinate ring of the affine cone of ${{\mathbb {C}}}[\hbox {Gr}(k,n)]$ is the quotient of the polynomial ring in generators $x_{i_1,\dots , i_k}$ with $1\le i_1<\dots \le i_k\le n$ by the ideal generated by the Plücker relations. For details: [23, Section 2]

### Cluster Algebra Structure for $\hbox {Gr}(2,n)$

As the first case to study, we fix $k=2$, where we can describe the Plücker relations more easily. This is a very classical case; it can be described using Ptolemy relations as we will see. Therefore, let $k=2$. Then, the Plücker relations are:

$$\begin{aligned} p_{i,j_0} p_{j_1,j_2}- p_{i,j_1}p_{j_0,j_2} + p_{i,j_2}p_{j_0,j_1}, \end{aligned}$$

where $1\le i\le n$, $1\le j_0<j_1<j_2\le n$. We can rewrite these as:

1.2 $$\begin{aligned} p_{ab}p_{cd} - p_{ac}p_{bd} + p_{ad}p_{bc} \quad \text{ for } \text{ all } a,b,c,d \text{ with } 1\le a<b<c<d\le n. \end{aligned}$$

#### Exercise 1.9

Check the above.

Using this and the facts from above, we get the following result:

#### Lemma 1.10

The homogenous coordinate ring of $\hbox {Gr}(2,n)$ is the quotient of the polynomial ring in variables $p_{ab}$, $1\le a<b\le n$, subject to the relations:

$$\begin{aligned} p_{ab}p_{cd} - p_{ac}p_{bd} + p_{ad}p_{bc} \quad \text{ for } \text{ all } 1\le a<b<c<d\le n. \end{aligned}$$

#### Remark 1.11

In this case, the Plücker coordinates can be parametrized by the diagonal and edges of a regular polygon $P_n$ with vertices $1,2,\dots , n$, say clockwise: the coordinate $p_{ab}$, $a<b$, corresponds to the diagonal or boundary edge connecting *a* and *b*. We can then interpret the relations (1.2) as “Ptolemy relations”, e.g., $p_{14}p_{26}=p_{12}p_{46}+p_{16}p_{24}$ in the example $(n=6)$:


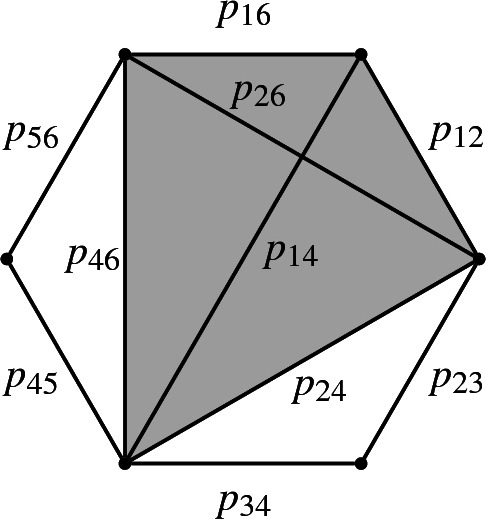


### The Quiver of a Triangulation of a Surface

We have seen that triangulations of convex polygons are important for the cluster algebra structure of $\hbox {Gr}(2,n)$. More generally, triangulations of surfaces give rise to cluster algebras and to cluster categories. An important notion is the quiver of such a triangulation. We explain here what we mean by a triangulation of a surface and how to find its quiver. The main reference for this part is [18].

Let *S* be a connected, oriented surface with boundary. Let $M\ne \emptyset$ be a finite set of marked points in ${\overline{S}}$. The points of *M* are on the boundary or in the interior of *S*. Assume that *M* contains at least one marked point on each boundary component and that (*S*, *M*) is not one of the following:

$$\begin{aligned} \left\{ \begin{array}{l} \text{ sphere } \text{ with } 1,2 \text{ or } 3 \text{ interior } \text{ points } \\ \text{ monogon } \text{ with } 0 \text{ or } 1 \text{ interior } \text{ points } \\ \text{ digon } \text{ or } \text{ triangle } \text{ with } \text{ no } \text{ interior } \text{ points }. \end{array} \right. \end{aligned}$$

We now restrict to the case where (*S*, *M*) has no punctures. Consider simple non-contractible arcs in (*S*, *M*), with endpoints in *M* (up to isotopy fixing endpoints). An **ideal triangulation** of (*S*, *M*) is a maximal collection of (isotopy classes of) such arcs which pairwise do not cross. Let *T* be an ideal triangulation of (*S*, *M*). Then, we associate with *T* a quiver $Q_T$ as follows.

The vertices of $Q_T$ are the arcs of *T* and the boundary segments. We draw an arrow $i\rightarrow j$ if *i* and *j* are arcs of a common triangle of *T* and *j* is clockwise from *i* (around a common endpoint) and if *i* and *j* are not both boundary segments. In the example, the quiver of a triangulation of a hexagon is drawn.

If *T* is a triangulation of a polygon $P_n$, like below, there are *n* boundary edges $(i,i+1)$. By definition of $Q_T$, there are no arrows between these. We will later consider quiver with arrows between vertices on the boundary. 
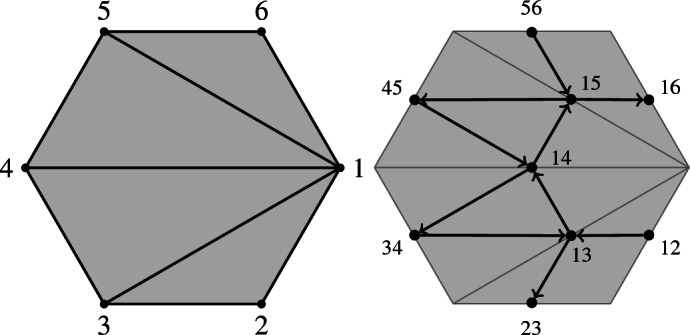


In these lectures, the surface is always a regular polygon $P_n$ and $M=\{1,2,\dots ,n\}$ the set of vertices of $P_n$. Therefore, for $Q_T$, the vertices are just the diagonals of the triangulation and the boundary edges of the polygon (sometimes called “frozen vertices”).

#### Theorem 1.12

[20, Proposition 12.6] Let $n\ge 5$. Let $P_n$ be a convex *n*-gon. The homogenous coordinate ring ${{\mathbb {C}}}[\hbox {Gr}(2,n)]$ of 2-planes in *n*-space is a cluster algebra:

$$\langle p_{ab}\mid 1\le a<b\le n\rangle /\{\hbox {Ptolemy relations}\}\otimes {{\mathbb {C}}}={{\mathbb {C}}}[\hbox {Gr}(2,n)]$$.

The cluster variables are the Plücker coordinates $p_{ab}$, where the (*a*, *b*) are the diagonals in $P_n$ and the coefficients are the Plücker coordinates $p_{12},p_{23},\dots , p_{n-1,n},p_{1n}$ (corresponding to the boundary edges of $P_n$).

The seeds are in bijection with the triangulations of $P_n$. The quiver of the seed is $Q_T$. Cluster mutation corresponds to the quadrilateral flip in a triangulation (and to the Ptolemy relations (1.2)

By the above result, ${{\mathbb {C}}}[\hbox {Gr}(2,n)]$ can be regarded as a cluster algebra of type $\hbox {A}_{n-3}$ with coefficients (cf. [27, Ex 8.2.3]). It is sometimes called **Ptolemy cluster algebra**.

#### Exercise 1.13

Find $Q_T$ for the triangulation *T* given by the diagonals $(13), (35), (36),(16),(17)$ of an octagon.


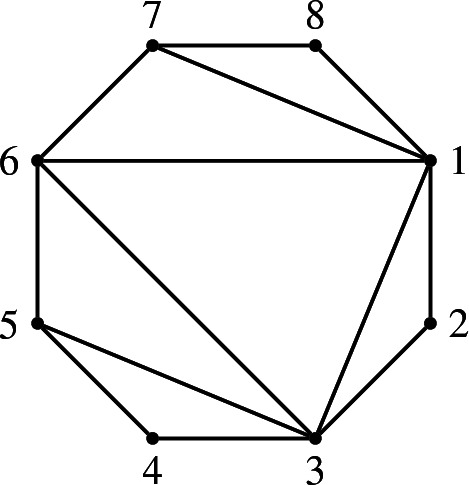


### Cluster Algebra Structure for $\hbox {Gr}(k,n)$

In this part, we recall how the Grassmannian $\hbox {Gr}(k,n)$ can be equipped with a cluster algebra structure. We will assume from now on that $k\le \frac{n}{2}$ as there is a canonical isomorphism $\hbox {Gr}(k,n)\cong \hbox {Gr}(n-k,n)$. To find a cluster algebra structure for arbitrary *k*, we will use the notion of a “strand diagram in a disk with *n* marked points” instead of a triangulation of a polygon. We write $S_n$ for the set of permutations of *n*. We write $D_n$ for a disk with *n* marked points $\{1,2,\dots , n\}$ on the boundary (going clockwise). The following definition is due to Postnikov [31].

#### Definition 1.14

Let $\sigma \in S_n$ be a permutation. An **alternating strand diagram** or **Postnikov diagram** **of type** $\sigma \in S_n$ in the disk $D_n$ with vertices $1,\dots , n$ is a collection of *n* oriented strands (smooth curves) $\gamma _1,\dots , \gamma _n$ (up to isotopy) in $D_n$ with $\gamma _i$ starting and *i* and ending at $\sigma (i)$ satisfying:

- The $\gamma _i$ have no self-intersections;
- There are finitely many intersections and they are transversal, of multiplicity 2;
- Crossings alternate (following any strand, the strands crossing it alternate between crossing from the left and crossing from the right);
- There are no “unoriented lenses”: if two strands cross, they form an oriented disk.

**Fig. 1:**
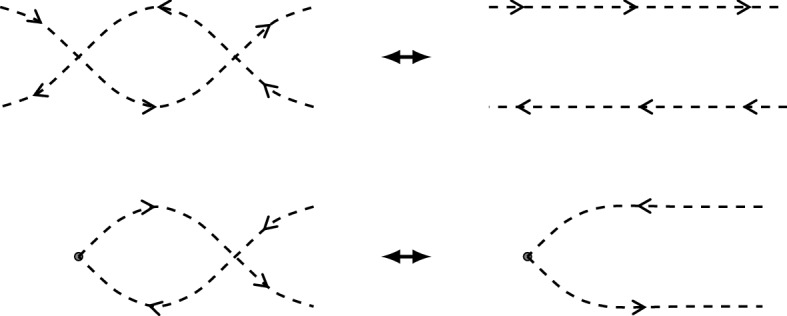
Untwisting and twisting moves in a Postnikov diagram

**Fig. 2:**
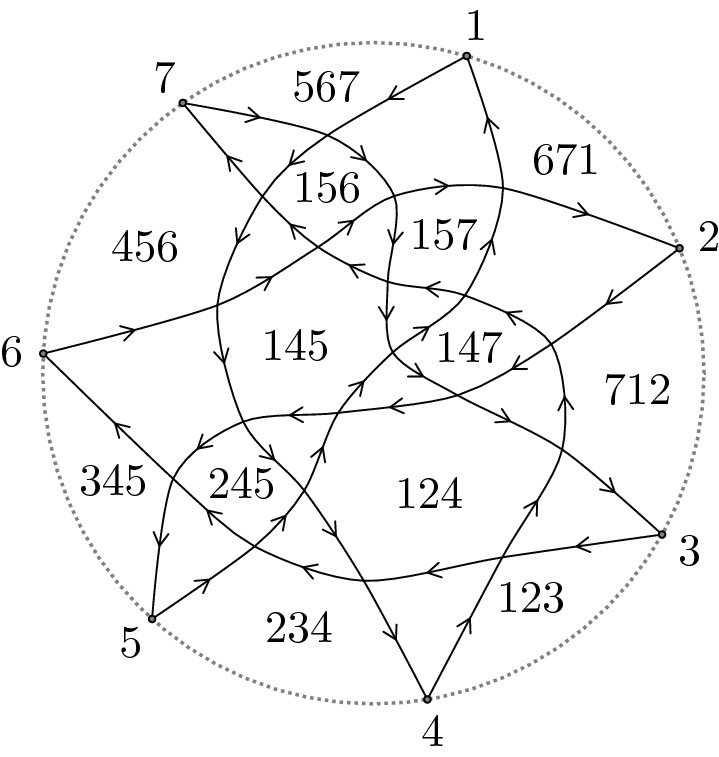
A $\sigma _{3,7}$ Postnikov diagram with its labels

For an illustration, we refer to Fig. 2. Postnikov diagrams can be simplified under two types of reductions as in Fig. 1, also called twisting and untwisting moves. The moves obtained by reflecting the diagrams of Fig. 1 in a horizontal line are also allowed. A Postnikov diagram is called **reduced** if it cannot be simplified with untwisting moves.

#### Remark 1.15

The conditions can be relaxed, i.e., if the surface has interior marked points or if it has several boundary components (like an annulus). Conditions (a) and (d) will no longer hold then. Strand diagrams appear as “webs” in [22]. Strand diagrams for orbifolds are introduced in [10].

Any Postnikov diagram divides the surface into alternating and oriented regions. We label the alternating regions by *i* whenever $\gamma _i$ is on the right of the region.

Let $\sigma _{k,n}\in S_n$ be the permutation $i\mapsto i+k$ (reducing modulo *n*).

#### Proposition 1.16

[31, Proposition 5]

(a) Any $\sigma _{k,n}$-diagram in $D_n$ has $k(n-k)+1$ alternating regions, $(k-1)(n-k-1)$ internal ones, *n* on the boundary.

(b) Each label is a *k*-subset of [*n*].

(c) Every *k*-subset of [*n*] appears as a label in a $\sigma _{k,n}$-diagram on $D_n$.

Note that if $\sigma =\sigma _{k,n}$, the *n* boundary alternating regions in (a) above have the labels [1, *k*], $[2,k+1]$, $\dots , [n,n+k]$ (reducing modulo *n*), see Fig. 2.

#### Example 1.17

Figure 2 shows an example of a Postnikov diagram of type $\sigma _{3,7}$ in $D_7$.

### The Quiver of a Postnikov Diagram

Postnikov diagrams of type $\sigma _{k,n}$ are instrumental in the cluster algebra structure of the Grassmannian. Each such diagram gives rise to a cluster, and the quiver of the latter determines the entire cluster algebra. We now recall how this quiver arises from the Postnikov diagram.

Let *D* be a $\sigma _{k,n}$-diagram. Each label of *D* gives a Plücker coordinate, so we can associated with *D* a collection of Plücker coordinates, $D\mapsto {\tilde{p}}(D)=p(D)\cup C$ where *C* are the Plücker coordinates of the boundary alternating regions, i.e., the $p_{i,i+1,\dots , i+k-1}$ for $i=1,\dots , n$. With this, we can associate a quiver *Q*(*D*) to *D*.

This is similar to quiver of a triangulation (use remark on bijection 1.21). The boundary convention is, however, different as we will see.

#### Definition 1.18

**Fig. 3:**
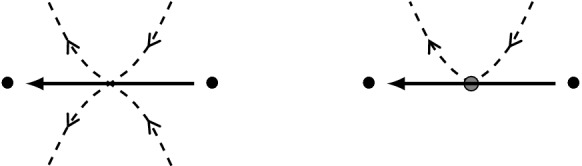
Orientation convention for the quiver *Q*(*D*), on the right for the boundary

Let *D* be a Postnikov diagram. The **quiver** *Q* **(***D***) of** *D* has as vertices the *k*-subsets of *D*. Its **frozen vertices** are the *k*-subsets of the boundary alternating regions of *D*. The arrows of *Q*(*D*) are given by the “flow”: whenever two *k*-subsets are separated by only two crossing strands, there is an arrow between them, following the orientation of the strands, see Fig. 3. At the end, we remove all 2-cycles that may have appeared through this.

#### Example 1.19

**Fig. 4:**
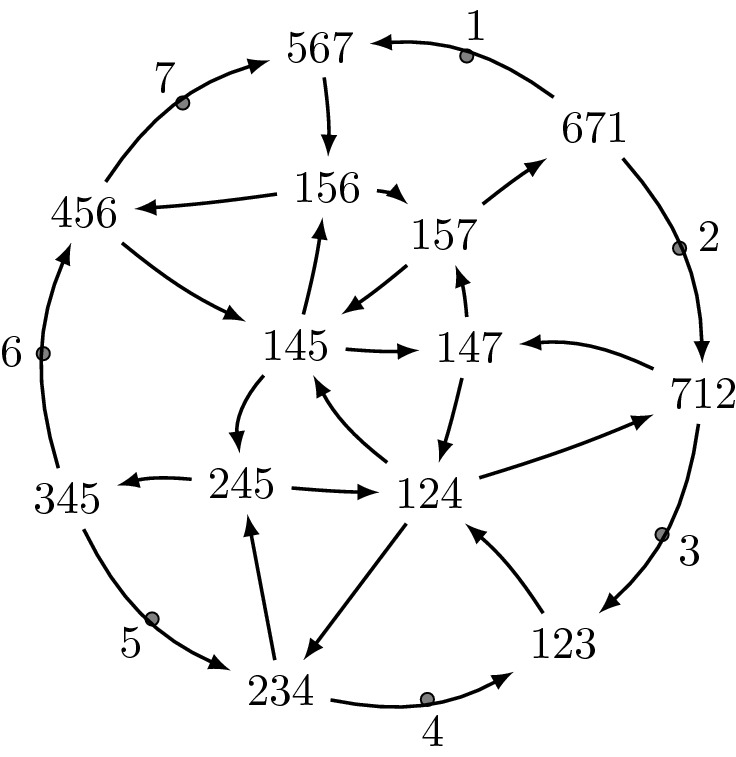
The quiver of the Postnikov diagram in Fig. 2

The quiver of the Postnikov diagram from Example 1.17 is in Fig. 4.

Note that the quiver of a Postnikov diagram contains arrows between boundary vertices, unlike the quiver of a triangulation.

#### Definition 1.20

To any Postnikov diagram *D* of type $\sigma _{k,n}$, one can define a cluster algebra: the initial seed is given by the set $\{x_I\}_{I\in {\tilde{p}}(D)}$ and the quiver *Q*(*D*). The cluster algebra ${{\mathcal {A}}}(D):={{\mathcal {A}}}(\{x_I\}_{I\in {\tilde{p}}(D)}, Q(D))$ is the ${{\mathbb {C}}}$-subalgebra of ${{\mathbb {C}}}((x_I)_I)$ generated by the $x_I$ with $I\in C$ and by the $x_I$, $I\in p(D)$, and all elements obtained from the latter under arbitrary sequences of mutations.

Each $\sigma _{k,n}$-diagram gives rise to a seed in ${{\mathcal {A}}}(D)$. Scott proves [32, Theorem 3] that there is an isomorphism $\varphi :{{\mathcal {A}}}(D){\mathop {\longrightarrow }\limits ^{\sim }}$ ${{\mathbb {C}}}[\hbox {Gr}(k,n)]$ sending $x_I$ to $p_I$ for any *k*-subset $I\in {\tilde{p}}(D)$. In other words, ${{\mathbb {C}}}[\hbox {Gr}(k,n)]$ can be viewed as a cluster algebra where each Plücker coordinate is a cluster variable and where the $\sigma _{k,n}$-diagrams give some of its seeds.

Note that here we do not invert coefficients (see [27, Section 9]). If we invert the coefficients, the cluster algebra is the coordinate ring of the Zariski-open subset of the Grassmannian defined by non-vanishing of the coefficients $p_{1,\dots , k}$, $p_{2,\dots , k+1}$, ... $p_{n,1,\dots , k-1}$.

#### Remark 1.21

**Fig. 5:**
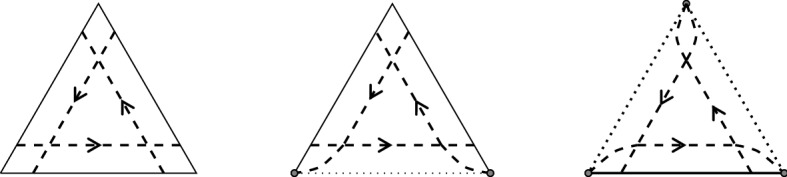
Modified version of Scott’s construction. The dotted lines indicate boundary edges

(Scott map) Let $k=2$ and $n\ge 4$. Then, there is a bijection between:

$$\begin{aligned} \begin{array}{ccc} \{ \text{ Triangulations } \text{ of } \text{ a } \text{ convex } n\text{-gon }\}&{\mathop {\longleftrightarrow }\limits ^{{1:1}}} \{\text{ reduced } \sigma _{2,n}\text{-diagrams }\} \end{array} \end{aligned}$$

arising from equipping each triangle in the triangulation with strand segments, as shown in Fig. 5, with a slight modification of Scott’s definition from [32, Section 3] in order for strands to end at the boundary vertices.

#### Exercise 1.22

Draw the $\sigma _{2,7}$-diagram *D* for the triangulation *T* from Exercise 1.13. Compare the two quivers $Q_T$ and *Q*(*D*).

#### Remark 1.23

A correspondence as the one given by Scott, see Remark 1.21, is not known for arbitrary *k*. The only other cases where a combinatorial construction is known are $\sigma _{k,n}$ diagrams for $n=2m$ and $k=m-1$; these arise from rhombic tilings [8].

By Remark 1.21, for $k=2$, the $\sigma _{2,n}$-diagrams correspond to triangulations of polygons and thus in this case, the Postnikov diagrams give all the seeds (Theorem 1.12). If *D* is $\sigma _{3,n}$-diagram for $n\in \{6,7,8\}$, then the cluster algebra ${{\mathcal {A}}}(D)$ of any $\sigma _{3,n}$-diagram is of finite type, i.e., there are only finitely many cluster variables. However, not all seeds ${{\mathcal {A}}}(D)$ arise from $\sigma _{3,n}$-diagrams.

In all other cases (with $k\le \frac{n}{2}$), the cluster algebra ${{\mathcal {A}}}(D)$ has infinitely many cluster variables.

## Dimer Models on Surfaces and Associated Algebras

By definition, the quiver of a $\sigma _{k,n}$-diagram is embedded in a disk. It has faces which are all oriented. This is the motivating example of dimer model with boundary, as we will see in Sect. 2.1. Any such dimer model gives rise to an algebra with relations which is strongly linked to a cluster-tilting object in the category ${\mathcal {F}}_{k,n}$, Sect. 2.2.

### Dimer Models with Boundary

Here, we recall the notion of a dimer model with boundary, one of the key tools for the endomorphism ring result in [7]. While we will only use the special cases of dimers from $\sigma _{k,n}$-diagrams, these quivers now appear much more widely.

#### Definition 2.1

1. A **dimer model (with boundary)** is a finite quiver *Q* that embeds into a surface *S*, such that each connected component of $S{\setminus } Q$ is simply connected and bounded by an oriented cycle.
2. The cycles bounding the connected components of $S{\setminus } Q$ are called the **unit cycles**. The arrows of *Q* are **internal** if they are contained in two faces and **boundary** if they are contained in one face. The vertices incident with boundary arrows are called **boundary vertices**.

In the case without boundary, the dimer model has been studied by various authors, e.g., [9, 11, 14]. The boundary convention has also been used independently in [6, 15, 17].

#### Example 2.2

The following quiver is an example of a dimer model with boundary, on a disk with 6 boundary vertices.


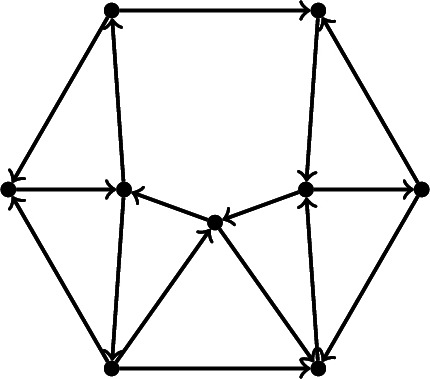


#### Example 2.3

(a) The quiver of any Postnikov diagram is a dimer model in the disk.

(b) Let *T* be a triangulation of a polygon $P_n$. If we complete the quiver $Q_T$ by $n-2$ clockwise and 2 anticlockwise arrows between the boundary vertices to close all the cycles involving boundary vertices, we get a dimer model in $P_n$. We denote the resulting quiver by *Q*(*T*).


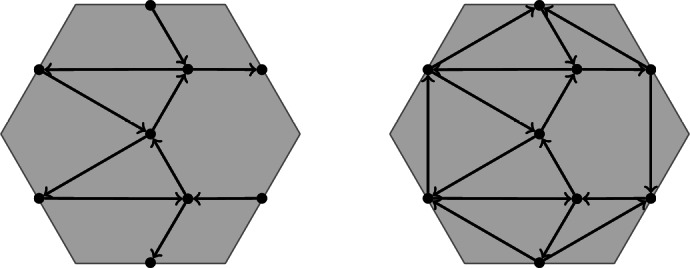


#### Exercise 2.4

Find a triangulation *T*, such that *Q*(*T*) as defined in Example 2.3 (b) is the dimer of Example 2.2.

### Algebras Associated with Dimer Models

We now want to associate algebras to dimer models. Most of the background for this section can be found in [7] and [25]. To be able to define these algebras, we first describe relations on paths which are inherited from the structure of the dimer model as a quiver with faces. If $\alpha$ is an internal arrow of a dimer model with boundary, then there are exactly two paths $\alpha ^{\pm }$ back from its head to its tail. For example, in the dimer model from Example 2.3 (b), for the arrow $\alpha :14\rightarrow 15$, the two paths back are $p_{\alpha }^+: (15\rightarrow 45)\circ (45\rightarrow 14)$ and $p_{\alpha }^-$ is $(15\rightarrow 16)\circ (16\rightarrow 12)\circ (12\rightarrow 13)\circ (13\rightarrow 14)$, composing arrows left to right: 
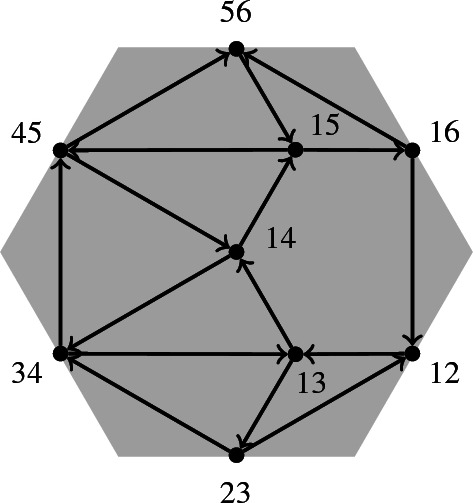


#### Definition 2.5

Let *Q* be a dimer model with boundary. The **dimer algebra** $A_Q$ **of** *Q* is quotient of the path algebra of ${{\mathbb {C}}}Q$ by the relations $p_{\alpha }^+=p_{\alpha }^-$ for every internal arrow of *Q*.

We may also take the completed version of this path algebra, see Remark 2.14.

Another way to describe the relations for the definition of the dimer algebra is as follows. Let *W* be the (natural) potential associated with *Q*:

$$\begin{aligned} W:=W(Q):=\sum _{ p \text{ pos. } \text{ unit } \text{ cycle }}p -\sum _{p \text{ neg. } \text{ unit } \text{ cycle }}p. \end{aligned}$$

Then, the relations $p_{\alpha }^+=p_{\alpha }^-$ arise from taking all cyclic derivatives $\partial (W)$ of *W* with respect to internal arrows.

Observe that $A_Q$ is an infinite-dimensional algebra.

#### Exercise 2.6

Any two unit cycles at a vertex of a dimer model *Q* commute. Why is this?

#### Remark 2.7

Let $i\in Q_0$ be a vertex of a dimer model. Let $U_i$ be a unit cycle at *i*. Then, $t:=\sum _{i\in Q_0} U_i$ is a central element of $A_Q$, since any two unit cycles at *i* commute. Therefore, we get ${{\mathbb {C}}}[t]\subseteq Z(A_Q)$.

Let $e_1,e_2,\dots , e_n$ be the idempotent elements of $A_Q$ corresponding to the boundary vertices of *Q* and let $e:=e_1+\dots +e_n$.

#### Definition 2.8

Let *Q* be a dimer model with boundary. The **boundary algebra** of *Q* is the idempotent subalgebra $B_Q:=eA_Qe$.

$B_Q$ has as basis all paths of *Q* between boundary vertices (up to the relations). Let $t:=U_1+\dots +U_n$ be the sum of the unit cycles at the boundary vertices. Then, ${{\mathbb {C}}}[t]\subseteq Z(B_Q)$.

Note that there is also a completed version of this, see Remark 2.14

#### Example 2.9

The boundary algebra from Example 2.2 is given by the following quiver:


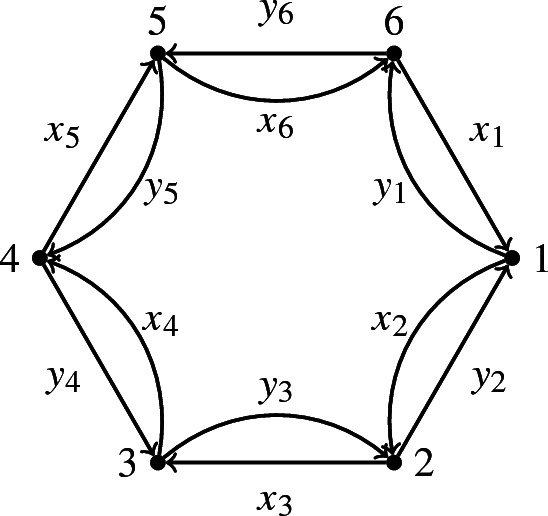


One can show that:

$$\begin{aligned} B_Q={{\mathbb {C}}}[x_i,y_i\mid i=1,\dots , n]/\left<\{xy-yx,x^2-y^4\}\right> \end{aligned}$$

and that $$Z(B_Q)={{\mathbb {C}}}[t]$$.

Note that $\{xy-yx,x^2-y^4\}$ is short for the 12 relations $x_iy_i-y_{i-1}y_{i-1}$, $x_ix_{i+1}-y_{i-1}y_{i-2}y_{i-3}y_{i-4}$ for $i_1,\dots , 6$ and where the indices are reduced modulo 6.

The boundary algebra $B_Q$ is also an infinite-dimensional algebra; in general, its global dimension is infinite. $A_Q$ and $B_Q$ are not well understood apart from the case where the surface *S* is a disk and the dimer models arise from Postnikov diagrams of type $\sigma _{k,n}$ or from the cases where *S* is a surface and *Q* arises from a triangulation of *S*.

From now on, we restrict to dimer models on disks. As we are interested in the cluster structure of the Grassmannian, we will also restrict the dimer models: we want them to correspond to $\sigma _{k,n}$-diagrams. Recall that any Postnikov diagram *D* on a disk determines a quiver *Q*(*D*) and that this is a dimer model with boundary. There is also a way to go from dimer models to Postnikov diagrams [31, Section 14]: for any arrow in the dimer model, draw two segments of oriented curves, crossing on the arrow, pointing in the same direction as the arrow, and then connect these; the strands correspond to zig-zag paths in the disk, cf. [11, Section 5].

#### Exercise 2.10

Find the Postnikov diagram for the dimer model above. Determine its permutation.

#### Definition 2.11

If *Q* is a dimer model on a disk, such that the associated Postnikov diagram is of type $\sigma _{k,n}$, we call *Q* a **(***k* **,** *n***)-dimer**.

#### Remark 2.12

- The (2, *n*)-dimers correspond to $Q_T$, *T* a triangulation of an *n*-gon.
- (*k*, *n*)-dimer exist for any (*k*, *n*). Examples of such arise from the rectangular $\sigma _{k,n}$-diagrams of Scott [32]

#### Theorem 2.13

**Fig. 6:**
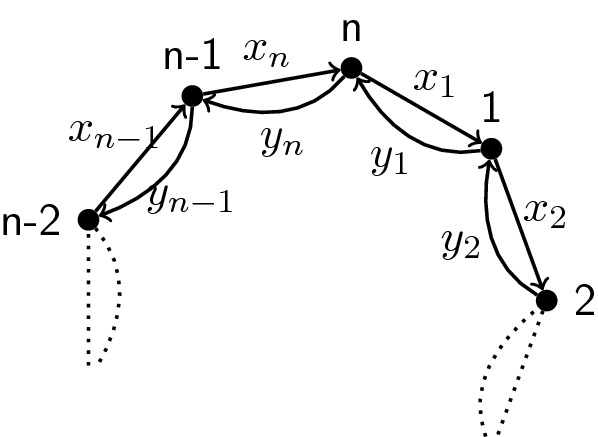
The quiver $$\Gamma _n$$

[7] Let *Q* and $Q'$ be two (*k*, *n*)-dimers. Let $e=e_1+\dots +e_n$ the sum of the boundary idempotents. Then:

$$\begin{aligned} eA_Qe\cong eA_{Q'}e\cong ({{\mathbb {C}}}\Gamma _n/\left<\{xy-yx,x^k-y^{n-k}\} \right>)^{op}, \end{aligned}$$

where $\Gamma _n$ is the quiver in Fig. 6 and the relations are with indices as in Example 2.9.

Let $B:=B_{k,n}:={{\mathbb {C}}}\Gamma _n/\left<\{xy-yx,x^k-y^{n-k}\} \right>$. Then, the boundary algebra of any (*k*, *n*)-dimer model is isomorphic to *B*.

#### Remark 2.14

The above theorem is about the completed versions of these algebras. For $A_Q$, the algebra ${\widehat{A}}_Q$ is the completion with respect to (*U*) for $U=\sum _{I\in Q_0} U_I$ and ${\widehat{B}}$ is the completion of *B* with respect to (*t*), $t=\sum _{i=1}^nx_iy_i$. Note that the completion ${\widehat{B}}$ of *B* with respect to (*t*) is the same as the completion with respect to the arrow ideal *m*, since $(m_{A_Q})^{N_1}\subseteq (U)\subseteq m_A$ and $(m_{B})^{N_2}\subseteq (t)\subseteq m_B$ for some $N_1,N_2$, [25, Section 3]. In particular, we have $e\widehat{A_Q}e\cong {\widehat{B}}$. See [7, Section 11].

The centers of *B* and ${\widehat{B}}$ are polynomial rings, $Z(B)={{\mathbb {C}}}[t]$ and $Z({\widehat{B}})={{\mathbb {C}}}[|t|]$.

We will from now on work with the completed versions as we want the Krull–schmidt Theorem to hold in the module categories. To simplify notation, we will write *B* and $A_Q$ instead of ${\widehat{B}}$ and $\widehat{A_Q}$ to simplify notation.

We consider *B*-modules which are free over the center and define:

$$\begin{aligned} {\mathcal {F}}_{k,n}:= & {} \hbox {CM}(B) := \{M B \hbox {-module}\mid M \text{ is } \text{ free } \text{ over } Z(B)\} \\= & {} \{M\mid \text{ Ext}^i_B(M,B)=0 \text{ for } \text{ all } i>0\}. \end{aligned}$$

Thee categories ${\mathcal {F}}_{k,n}$ have been introduced by Jensen–King–Su. In [25], the authors prove that ${\mathcal {F}}_{k,n}$ provides an additive categorification of Scott’s cluster algebra structure on the Grassmannian.

## Grassmannian Cluster Categories

In this section, we study the Grassmannian cluster categories in more detail. As before, we always assume $k\le \frac{n}{2}$. It is our goal to understand the categories ${\mathcal {F}}_{k,n}$ better, for example, by giving a description of (some of) their indecomposable modules. We start by describing the building blocks for these categories, the so-called rank one modules, Sect. 3.1. Then, we show how dimer models can be used as an approach to cluster categories, Sect. 3.2. In general, the category ${\mathcal {F}}_{k,n}$ has infinitely many indecomposable objects. In Sect. 3.3, we recall the known results for the finite types. Then, we concentrate on the infinite types, Sect. 3.4. In the Grassmannian cluster categories, all objects are periodic under the Auslander–Reiten translate, as we recall in Sect. 3.5. In the last three parts, we first describe a link to certain root systems, Sect. 3.6, give a construction of rank 2 modules, Sect. 3.7, and then show how the Plücker coordinates or the Grassmannian cluster categories ${\mathcal {F}}_{k,n}$ give rise to friezes, Sect. 3.8.

### Rank 1 Modules

The category ${\mathcal {F}}_{k,n}$ does not contain the simple *B*-modules at the vertices as they are not free over the center. However, there is a class of objects in these categories which is well understood and whose elements serve as a building blocks for arbitrary modules, namely the rank 1 modules. The **rank** of a module $M\in {\mathcal {F}}_{k,n}$ is defined to be $\frac{1}{n}\text{ rk}_ZM$. In other words, when restricting *M* to the center of *B*, at each vertex of the quiver of *B*, the module has a fixed number of copies of *Z* and this number is the rank of *M*. Among the indecomposable modules, the rank 1 modules play a special role as they appear in filtrations of higher rank modules. As is shown in [25, Section 5], there is a bijection between indecomposable rank 1 modules in ${\mathcal {F}}_{k,n}$ and *k*-subsets of $\{1,2,\dots , n\}$. As we know, these are in bijection with Plücker coordinates and hence with certain cluster variables, see Sect. 1.4. We write ${{\mathbb {M}}}_I$ for the indecomposable rank 1 module with *k*-subset *I* of [*n*].

We first describe how *B* acts on the rank 1 modules and then show how these modules can be understood as a lattice diagram with vertices for basis elements and arrows for the actions of the $x_i$, $y_i$. Note that all modules in ${\mathcal {F}}_{k,n}$ are infinite-dimensional as they are free over the center.

Any rank 1 module is given by *n* copies of the center, $U_1,\dots , U_n$, with $U_i:={{\mathbb {C}}}[|t|]$. Consider ${{\mathbb {M}}}_I$, *I* a *k*-subset of [*n*]. The actions of $x_i$ and of $y_i$ on the $U_i$ are as follows:

$$\begin{aligned} x_i:U_{i-1}\rightarrow U_i \text{ acts } \text{ as } \left\{ \begin{array}{ll} 1 &{}\;\; \hbox {if}\, i\in I \hbox {} \\ t &{}\;\; \text{ if }\, i\notin I \end{array}\right. y_i:U_{i}\rightarrow U_{i-1} \text{ acts } \text{ as } \left\{ \begin{array}{ll} t &{}\;\; \hbox { if}\ i\in I \\ 1 &{}\;\; \text{ if } \,i\notin I. \end{array}\right. \end{aligned}$$

We can view this as a lattice diagram on an infinite cylinder. For example, for $k=3$ and $n=7$, $I=\{2,4,5\}$. The arrows $x_2,x_4,x_5$ and the arrows $y_1,y_3,y_6,y_7$ all act as multiplication by 1, the other arrows as multiplication by *t*. 
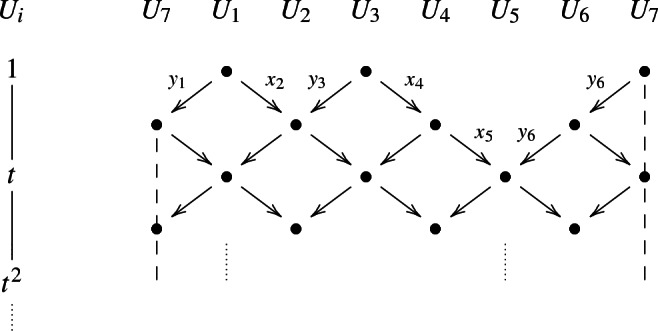


The **rim of a rank 1 module** ${{\mathbb {M}}}_I$ is formed by the top vertices in its lattice diagram and by the arrows connecting them. If the rim of ${{\mathbb {M}}}_I$ has two successive arrows $y_i$, $x_{i+1}$ for some *i*, we say that the rim or ${{\mathbb {M}}}_I$ has a **peak (at** *i*).

#### Remark 3.1

In ${\mathcal {F}}_{2,n}$ every indecomposable object is a rank 1 module, i.e., of the form ${{\mathbb {M}}}_{i,j}$ for some $1\le i\ne j\le n$. The objects ${{\mathbb {M}}}_{i,i+1}$ are the indecomposable projective–injective objects.

#### Exercise 3.2

Show that $\text{ Hom}_M({{\mathbb {M}}}_I,{{\mathbb {M}}}_J)\cong {{\mathbb {C}}}[|t|]$ for all *I*, *J*.

#### Definition 3.3

Let *I* and *J* be *k*-subsets of [*n*]. We say that *I* and *J* **cross** if the complete graph $K_{I{\setminus } J}$ on $I{\setminus } J$ intersects $K_{J{\setminus } I}$.

Note that *I* and *J* do not cross if and only if the two *k*-subsets appear together in a $\sigma _{k,n}$-diagram. One can prove that the maximal non-crossing collections are exactly the ones arising from $\sigma _{k,n}$-diagrams: by [32, Corollary 1], the *k*-subsets of any $\sigma _{k,n}$-diagram form a maximal non-crossing collection, and by [30, Theorem 7.1], every such collection arises in this way.

For an example of two crossing 4-subsets of [8], consider $I=\{1,4,6,8\}$ and $J=\{2,5,6,7\}$: 
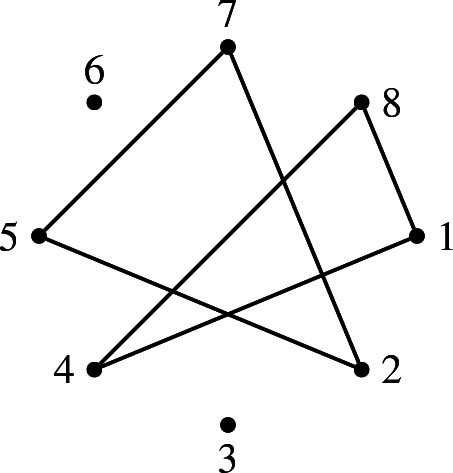


Crossing subsets are exactly the ones giving rise to non-trivial extensions:

#### Proposition 3.4

[25, Proposition 5.6] $\hbox {Ext}^1_B({{\mathbb {M}}}_I,{{\mathbb {M}}}_J)=0$ if and only if *I* and *J* do not cross.

One can use this to find cluster-tilting objects in $\mathcal F_{k,n}$: let *Q* be a (*k*, *n*)-dimer and let $T:=\oplus _{I\in Q_0}{{\mathbb {M}}}_I$. Then, *T* is a maximal rigid object in ${\mathcal {F}}_{k,n}$ [7, 25].

### Dimer Models as Combinatorial Approach to Cluster Categories

Here, we recall the main result of [7] and sketch its proof. This result tells us that any $\sigma _{k,n}$ gives rise to an endomorphism algebra of a cluster-tilting object and that, in particular, we can obtain the category ${\mathcal {F}}_{k,n}$ from this by going to the boundary algebra. Theorem 2.13 above is a consequence of the following result:

#### Theorem 3.5

[7, Theorem 10.3] Let *Q* be a (*k*, *n*)-dimer and *B* as above, $e=e_1+\cdots +e_n$ the sum of the idempotents for the boundary vertices of *Q*. Then,

$$\begin{aligned} A_Q\cong \text{ End}_B(T), \end{aligned}$$

and hence, $$eA_Qe\cong B^{op}$$.

#### Proof (Sketch of proof)

We define a map:

$$\begin{aligned} \begin{array}{lll} A_Q &{}{\mathop {\rightarrow }\limits ^{g}} &{} \text{ End}_B(T)=\text{ Hom}_B(\oplus _{I\in Q_0}{{\mathbb {M}}}_I,\oplus _{I\in Q_0}{{\mathbb {M}}}_I) \\ e &{} \mapsto &{} \text{ id}_{{{\mathbb {M}}}_I} \\ \alpha :I\rightarrow J &{} \mapsto &{} \varphi _{IJ}:{{\mathbb {M}}}_I\rightarrow {{\mathbb {M}}}_J (``\text{ minimal } \text{ codimension } \text{ map }'') \end{array} \end{aligned}$$

and extend to *T* (by 0’s).

- One shows that *g* is an algebra homomorphism.
- For the surjectivity of *g*: to find $g^{-1}(\varphi _{IJ})$, we need a “minimal” path $p_{IJ}:I \rightarrow \cdots \rightarrow J$ in *Q* (unique in $A_Q$). It is difficult to see that this maps to $\varphi _{IJ}$. For this, we use weights on the arrows of *Q*, these are subsets of [*n*], and show that the minimal path avoids at least one label of [*n*] (using the proper ordering of strands around vertices).
- For the injectivity of *g*, we prove a Lemma stating that if $p:I\rightarrow J$ is a path in *Q*, then there exists $r\ge 0$, such that $p=u^r\circ p_{IJ}$ where *u* is any unit cycle at *J*. This yields injectivity: take $m\in A_Q$ with $g(m)=0$. Without loss of generality, $m=e_IA_Qe_J$. By the lemma, $m=\sum _{r=0}^l\lambda _r u^rp_{IJ}$ for some coefficients $\lambda _r$, and so, $g(m)=\sum _{r=0}^l\lambda _r u^r\varphi _{IJ}=0$. Since the $t^r\varphi _{IJ}$ are linearly independent elements of $\text{ Hom}_B({{\mathbb {M}}}_I,{{\mathbb {M}}}_J)$, this implies $\lambda _r=0$ for all *r*.
- For the statement $eA_Qe\cong B^{op}$, use the isomorphism in the theorem: $eA_Qe\cong g(e)\text{ End}_B(T)g(e)=\text{ End}_BP=B^{op}$ where *P* is the sum of the *n* indecomposable projective–injective modules.

$$\square $$

### Grassmannian Cluster Categories of Finite Type

The category ${\mathcal {F}}_{k,n}$ has of finitely many indecomposables if and only if $k=2$ or $k=3$ and $n\in \{6,7,8\}$. To visualize this, we use the Auslander–Reiten quiver of ${\mathcal {F}}_{k,n}$. It has as vertices the isomorphism classes of indecomposable objects and it has an arrow for every irreducible map. The dotted lines in the Auslander–Reiten quiver are the Auslander–Reiten translate, sending an object to its neighbor on the left. The Auslander–Reiten quiver provides a good understanding of the category, since the ${\mathcal {F}}_{k,n}$ are Krull–Schmidt, every object can be uniquely written as a direct sum of indecomposables. In the case $k=2$, the category ${\mathcal {F}}_{2,n}$ is a cluster category of type $\hbox {A}_{n-3}$ (with projective–injective objects). Its Auslander–Reiten quiver sits on a Moebius strip. Recall that for ${\mathcal {F}}_{2,n}$, all the indecomposables are of the form ${{\mathbb {M}}}_{i,j}$ (Remark 3.1).

#### Example 3.6

The Auslander–Reiten quiver of ${\mathcal {F}}_{2,6}$ has the following form:There are $6\atopwithdelims ()2$ indecomposable modules, indexed by the 2-subsets of 6. The projective–injective indecomposables are the ${{\mathbb {M}}}_{i,i+1}$ (reducing modulo 6), drawn in a box in the quiver.

The Auslander–Reiten quivers of the categories ${\mathcal {F}}_{3,n}$ have been described in [25, Figures 10,11,12]. We recall them here. Some of the indecomposable objects are rank 1 modules, but there are also rank 2 modules ($n=6,7,8$) and rank 3 modules ($n=8$).

#### Example 3.7

Auslander–Reiten quiver of ${\mathcal {F}}_{3,6}$. It is formed by 4 slices of shape $D_4$and six additional vertices, corresponding to the projective–injective modules ${{\mathbb {M}}}_{i,i+1,i+2}$ (reducing indices modulo 6):There are ${6 \atopwithdelims ()3}=20$ rank 1 indecomposables and two rank 2 indecomposables, the latter indicated by a vertex $\bullet$ (the left most and the right most are identified). The projective–injectives are drawn in boxes.

#### Example 3.8

The Auslander–Reiten quiver of ${\mathcal {F}}_{3,7}$ is formed by 7 slices of shape $E_6$:and 7 additional vertices, corresponding to the projective–injective modules ${{\mathbb {M}}}_{i,i+1,i+2}$ (reducing modulo 7). This figure shows part of it. To complete it, one can continue along the dotted lines, using the fact that $\tau ^{-2}({{\mathbb {M}}}_{i,j,k})={{\mathbb {M}}}_{i+3,j+3,k+3}$:There are ${7 \atopwithdelims ()3}=35$ rank 1 indecomposables and 14 rank 2 indecomposables, indicated by $\bullet$.

#### Example 3.9

The Auslander–Reiten quiver of ${\mathcal {F}}_{3,8}$ is formed by 16 slices of shape $E_8$:and 8 additional vertices, corresponding to the projective–injective modules ${{\mathbb {M}}}_{i,i+1,i+2}$ (reducing modulo 8), drawn in boxes. Apart from the rank 1 modules, there are rank 2 modules drawn as $\bullet$ and rank 3 modules drawn as $\blacksquare$. The rest of the shape of the Auslander–Reiten quiver can be obtained using the fact that for rank 1 modules, $\tau ^{-2}({{\mathbb {M}}}_{i,j,k})={{\mathbb {M}}}_{i+3,j+3,k+3}$:There are ${8\atopwithdelims ()3}=56$ rank 1 indecomposables, 56 rank 2 indecomposables, and 24 rank 3 indecomposables.

### Structure of ${\mathcal {F}}_{k,n}$ in Infinite Types

The aim of the remainder of these notes is to provide more information about the categories ${\mathcal {F}}_{k,n}$ which have infinitely many indecomposables. We will describe part of the Auslander–Reiten quiver in the general situation. The main tool in this section is a result determining Auslander–Reiten sequences.

#### Remark 3.10

(i) If $M\in {\mathcal {F}}_{k,n}$ is indecomposable rigid, then it has a filtration $$M\cong \begin{array}{c}{{\mathbb {M}}}_{I_1} \\ \vdots \\ {{\mathbb {M}}}_{I_d}\end{array}$$ by rank 1 modules ${{\mathbb {M}}}_{I_1},\dots , {{\mathbb {M}}}_{I_d}$ and this filtration is unique. The rank of *M* is *d*.
(ii) Note that the rank is additive on Auslander–Reiten sequences.

There are certain canonical Auslander–Reiten sequences which involve rank 1 modules. If ${{\mathbb {M}}}_I$ is an indecomposable whose rim has *s* peaks (see Sect. 3.1), then $\Omega ({{\mathbb {M}}}_I)$ is a rank $(s-1)$ module [2], where $\Omega$ is the syzygy functor. In particular, if $I=\{i,j,j+1,\dots , j+k-2\}$ with $i+1\ne j$ and $i-1\ne j+k-2$, the rim of ${{\mathbb {M}}}_I$ has two peaks and $\Omega ({{\mathbb {M}}}_I)={{\mathbb {M}}}_J$ is also a rank 1 module, with $J=\{i+1,i+2,\dots , i+k-1,j+k-1\}$, cf. [2, Section 2].

#### Theorem 3.11

[3] Let $3\le k\le \frac{n}{2}$ and $I=\{i,j,j+1,\dots , j+i-2\}$ and *J*, such that $\Omega ({{\mathbb {M}}}_I)={{\mathbb {M}}}_J$. Then, there exists an Auslander–Reiten sequence:

$$\begin{aligned} {{\mathbb {M}}}_I\hookrightarrow \frac{{{\mathbb {M}}}_X}{{{\mathbb {M}}}_Y} \twoheadrightarrow {{\mathbb {M}}}_J \end{aligned}$$

with rigid middle term $\frac{{{\mathbb {M}}}_X}{{{\mathbb {M}}}_Y}$ for $X=\{i+1,j,\dots , j+k-3,j+k-1\}$ and $Y=I\cup J{\setminus } X$.

The middle term is indecomposable if and only if $j\ne i+2$ and if $j=i+2$, $\frac{{{\mathbb {M}}}_X}{{{\mathbb {M}}}_Y}=P_i\oplus {{\mathbb {M}}}_{\{i,i+2,\dots , i+k-1,i+k+1\}}$ for $P_i={{\mathbb {M}}}_{\{i+1,i+2,\dots , i+k\}}$.

#### Remark 3.12

- To prove the rigidness of the middle term, one shows that $\dim$Ext$^1({{\mathbb {M}}}_I,{{\mathbb {M}}}_J)=1$ (using the description of extensions between rank 1 modules from [2]).
- In case $k=3$, the theorem above covers all Auslander–Reiten sequences where both end terms are rank 1 modules.

#### Remark 3.13

For $(k,n) \in \{(3,9),(4,8)\}$, the category ${\mathcal {F}}_{k,n}$ is of infinite type but tame (i.e., these are the first infinite cases to study). These are tubular categories: their Auslander–Reiten quiver is formed by tubes. Using Theorem 3.11 and computing syzygies for rank 2 modules, we are able to determine the tubes with low rank rigid modules [3]. The tubes for $\mathcal F_{3,9}$ are of rank 2, 3, 6 and ${\mathcal {F}}_{4,8}$ of rank 2, 4, 4.

### Periodicity in ${\mathcal {F}}_{k,n}$

While the categories ${\mathcal {F}}_{k,n}$ are not well understood in general, we know that all its objects are periodic under the Auslander–Reiten translate, as we recall now. For any *k*-subset *I* of [*n*], $I=\{i_1,i_2,\dots , i_k\}$, we define $I+k$ to be the *k*-subset $\{i_1+k,i_2+k,\dots , i_k+k\}$. Let $\tau$ be the Auslander–Reiten translate in ${\mathcal {F}}_{k,n}$.

#### Example 3.14

Let ${{\mathbb {M}}}_I$ be an indecomposable in ${\mathcal {F}}_{k,n}$. Then: $\Omega ^2({{\mathbb {M}}}_I)={{\mathbb {M}}}_{I+k}$.

#### Proposition 3.15

Every indecomposable in ${\underline{{\mathcal {F}}_{k,n}}}$ is $\tau$-periodic with period a factor of $\frac{2}{k}\hbox {lcm}(k,n)$.

The proof of this proposition uses ${\mathcal {F}}_{k,n}\simeq$ CM$^{{{\mathbb {Z}}}_n}(R_{k,n})$ (graded Morita equivalence) for the ring $R_{k,n}:={{\mathbb {C}}}[x,y]/(x^k-y^{n-k})$ where *x* has degree 1 and *y* has degree $-1$, cf. [15, Theorem 3.22]. Alternatively, one can use [26, Theorem 8.3] to find $\tau ^n(M)=M$ for any indecomposable *M* in ${\underline{Fk}}$.

#### Remark 3.16

The quivers of the cluster-tilting objects that are given by Scott’s quadrilateral arrangements have the following shapes. If we write $T=T'\oplus P_1\oplus P_2 \oplus \dots \oplus P_n$ where the $P_i$ are projective–injective, then the quiver of the endomorphism algebra of $T'$, for $T'$ arising from the quadrilateral arrangement, is as on the left, with $k-1$ rows and $n-k-1$ columns. It can be mutated to the quiver on the right:These quivers are also appear in Keller’s work [26] on the periodicity conjecture. In terms of these quivers, ${\mathcal {F}}_{k,n}$ has finite representation type if and only if it has a cluster-tilting object $T=T'\oplus (\oplus _i P_i)$, such that the quiver of the endomorphism algebra of $T'$ is a linear orientation of $\hbox {A}_n$ or it is formed by 2, 4, or 6 oriented triangles forming one rectangle with side lengths 2 and 2, 3, or 4.

#### Remark 3.17

The periodicity is a first approach to the Auslander–Reiten quiver of the category ${\mathcal {F}}_{k,n}$. We claim that, in fact, this category is tubular in all infinite types (work in progress with Bogdanic, Garcia Elsener, and Li), even beyond the tame cases, cf. Remark 3.13.

### Root Systems Associated with ${\mathcal {F}}_{k,n}$

In this part, we describe a link from the category ${\mathcal {F}}_{k,n}$ to a certain root system. While in the finite types, this link is well described, in general, the connection remains mysterious. The main references for this section are [25, Section 8] and [3, Section 2].

Consider the graph:with nodes $1,2,\dots , n-1$ on the horizontal line and node *n* branching off from node *k*. To this graph, a root system $\Phi _{k,n}$ is associated, each node corresponds to a simple root, and edges indicate simple roots which can be added. The root system of $J_{k,n}$ has simple roots $\alpha _i:=-e_i+e_{i+1}$ for $i=1,\dots , n-1$ and $\beta =e_1+\dots + e_k$ for $e_1,\dots , e_n$ the standard basis vectors of ${{\mathbb {C}}}^n$. Note that if $k=2$, $J_{2,n}$ is a Dynkin diagram of type $\hbox {D}_n$. If $k=3$ and $n\in \{6,7,8\}$, the graph $J_{3,n}$ is a Dynkin diagram of type $\hbox {E}_6$, $\hbox {E}_7$, or $\hbox {E}_8$, respectively.

Let ${{\mathbb {Z}}}^n(k):=\{{\underline{x}} \in {{\mathbb {Z}}}^n\mid k \text{ divides } \sum _i x_i\}$. The root system $\Phi _{k,n}$ of $J_{k,n}$ can be identified with ${{\mathbb {Z}}}^n(k)$ via $\alpha _i \leftrightarrow -e_i+e_{i+1}$, for $i\le n-1$ and $\beta \leftrightarrow e_1+\dots +e_k$.

Define $q:{{\mathbb {Z}}}^n(k)\rightarrow {{\mathbb {Z}}}$ to be $q({\underline{x}})=\sum _{i=1}^n x_i^2 + \frac{2-k}{k^2}(\sum _{i=1}^n x_i)^2$.

Then, the roots for $J_{k,n}$ correspond to the vectors ${\underline{a}}$ of ${{\mathbb {Z}}}^n(k)$ with $q({\underline{a}})\le 2$. The vectors with $q({\underline{a}})=2$ are the **real** roots, and the vectors with $q({\underline{a}})<2$ are **imaginary roots**. The **degree of a root** $\gamma$ is its coefficient at $\beta$: if $\gamma =\sum _{i=1}^{n-1}m_i\alpha _i + m\beta$, $m_i,m\in {{\mathbb {Z}}}$, then $\deg \gamma =m$.

In Section 8, [25] define a map ind$\,{\mathcal {F}}_{k,n}{\mathop {\longrightarrow }\limits ^{\varphi }} \Phi _{k,n}$. The map $\varphi$ associates with each indecomposable a positive root of $\Phi _{k,n}$.

Let *M* be an indecomposable rank *d* module. Assume that *M* has a filtration by rank 1 modules, $$M=\begin{array}{c}{{\mathbb {M}}}_{I_1} \\ \hline \vdots \\ \hline {{\mathbb {M}}}_{I_d} \end{array}$$ for *k*-subsets $I_1,\dots , I_d$. For $i=1,\dots , n$, let $a_i$ be the multiplicity of *i* in $I_1\cup \dots \cup I_d$. We associate with *M* the vector ${\underline{a}}(M)=(a_1,\dots , a_n)$. Then, $\varphi (M)$ is defined to be the root corresponding to $(a_1,\dots , a_n)\in {{\mathbb {Z}}}^n(k)$.

#### Example 3.18

Let $n=6$ and $k=3$. Therefore, $J_{3,6}$ is as follows:Let $I=\{1,3,5\}$ and $J=\{2,4,6\}$.

1. $$M:={{\mathbb {M}}}_I$$: $${\underline{a}}=(1,0,1,0,1,0)$$ and $$\varphi (M)=\beta +\alpha _2+\alpha _3+\alpha _4$$.
2. Let $M={{\mathbb {M}}}_I/{{\mathbb {M}}}_J$. Then, ${\underline{a}}=(1,1,1,1,1,1)$ which corresponds to $2\beta + \alpha _1+2\alpha _2 + 3\alpha _3 + 2\alpha _4+\alpha _5$, the highest root for $\hbox {E}_6$.

#### Exercise 3.19

Compute $\varphi (M)$ for $M={{\mathbb {M}}}_J$ and for $M={{\mathbb {M}}}_J/{{\mathbb {M}}}_I$ from Exercise 3.18.

#### Remark 3.20

Let *M* be indecomposable with filtration $M={{\mathbb {M}}}_{I_1}/{{\mathbb {M}}}_{I_2}/\dots /{{\mathbb {M}}}_{I_d}$. Then, one observes that $\varphi (M)$ is a root of degree *d*.

#### Question 1

What is the connection between indecomposable rank *r*-modules in ${\mathcal {F}}_{k,n}$ and roots for $J_{k,n}$? What is the connection between rigid indecomposables in ${\mathcal {F}}_{k,n}$ and real roots for $J_{i,n}$?

For $r=1$, there is a bijection:

$$\begin{aligned} \left\{ \text{ indecomposable } \text{ rank } 1\text{-modules }\}/_\sim \quad {\mathop {\longleftrightarrow }\limits ^{1:1}} \quad \{ \text{ real } \text{ roots } \text{ for } J_{k,n} \text{ of } \text{ degree } \text{1 }\right\} . \end{aligned}$$

In finite types, one finds:

$$\begin{aligned} \left\{ \text{ indecomposable } \text{ rank } r\text{-modules }\}/_\sim \quad {\mathop {\longleftrightarrow }\limits ^{r:1}} \quad \{ \text{ real } \text{ roots } \text{ for } J_{k,n} \text{ of } \text{ degree } r\right\} \end{aligned}$$

as in these types, the higher rank modules “cycle”: let $k=3$ and $n\in \{6,7,8\}$, and then, the indecomposables are all of rank $\le 3$. And $M={{\mathbb {M}}}_I/{{\mathbb {M}}}_J$ is an indecomposable rank 2 module if and only if ${{\mathbb {M}}}_J/{{\mathbb {M}}}_I$ is indecomposable. Furthermore, $\varphi ({{\mathbb {M}}}_I/{{\mathbb {M}}}_J)=\varphi ({{\mathbb {M}}}_J/{{\mathbb {M}}}_I)$, cf. Exercise 3.19 for $n=6$. For the rank 3 modules, $n=8$, we have ${{\mathbb {M}}}_I/{{\mathbb {M}}}_J/{{\mathbb {M}}}_L$ is indecomposable if and only if ${{\mathbb {M}}}_J/{{\mathbb {M}}}_L/{{\mathbb {M}}}_I$ is indecomposable if and only if ${{\mathbb {M}}}_L/{{\mathbb {M}}}_I/{{\mathbb {M}}}_J$ is indecomposable, all with the same root in $\Phi _{3,8}$.

The first cases that are not fully understood are the tame cases ${\mathcal {F}}_{3,9}$ and ${\mathcal {F}}_{4,8}$, cf. Remark 3.13. It is known that in these cases, every real root of degree 2 yields exactly two rigid indecomposable rank 2 modules. However, there exist rigid indecomposable rank 2 modules in ${\mathcal {F}}_{4,8}$ with associated imaginary root. See Example 3.21 and Exercise 3.22.

#### Example 3.21

Let $n=8$, $k=4$, $I=\{2,5,6,8\}$, and $J=\{1,3,4,7\}$. The module ${{\mathbb {M}}}_I/{{\mathbb {M}}}_J$ is rigid indecomposable, cf. [25, Figure 13]. The lattice diagram of ${{\mathbb {M}}}_I/{{\mathbb {M}}}_J$ is below—vertices indicated with $\bullet$ correspond to one-dimensional vector spaces and vertices indicated with $\diamond$ correspond to two-dimensional vector spaces and arrows $=>$ to maps between two-dimensional vector spaces. The module ${{\mathbb {M}}}_J$ is visible as the submodule on the boxed vertices. One can check that the associated root is imaginary:Note that ${{\mathbb {M}}}_J/{{\mathbb {M}}}_I$ is not indecomposable.

#### Exercise 3.22

Find $\varphi ({{\mathbb {M}}}_I/{{\mathbb {M}}}_J)$ for *I*, *J* as in Example 3.21. Compute $q({\underline{a}}({{\mathbb {M}}}_I/{{\mathbb {M}}}_J))$.

### Rank 2 Modules in ${\mathcal {F}}_{k,n}$

To describe the category ${\mathcal {F}}_{k,n}$, we have as a first tool the rank 1 modules. In the finite types, we also have examples of rank 2 and rank 3 modules, as seen in Sect. 3.3. Here, we give a construction for rank 2 modules in general. Let *I* and *J* be two *k*-subsets. Assume that *I* and *J* are tightly 3-interlacing, i.e., that $|I{\setminus } J|=|J{\setminus } I|=3$ and that the non-common elements of *I* and *J* interlace. We want to define a rank 2 module ${{\mathbb {M}}}(I,J)$ in similar way as rank 1 modules are defined. Let $V_i:={{\mathbb {C}}}[|t|]\oplus {{\mathbb {C}}}[|t|]$, $i=1,\dots , n$. We will need to say how $x_i$, $y_i$ act. For this, define matrices $$M(c)=\begin{pmatrix} t &{} c \\ 0 &{} 1 \end{pmatrix}$$ and $$N(c)=\begin{pmatrix} 1 &{} -c \\ 0 &{} t \end{pmatrix}$$ with $c\in {{\mathbb {C}}}$ and $$D_1=\begin{pmatrix} 1 &{} 0 \\ 0 &{} 1 \end{pmatrix}=\hbox {id}$$, $D_2=t\hbox {id}$. These matrices give rise to factorisations of $t\hbox {id}$, and we have $M(c)N(c)=N(c)M(c)=t\hbox {id}$ for all *c*.

#### Example 3.23

Let $k=3$, $n=6$, $I=\{2,4,6\}$, and $J=\{1,3,5\}$. We define ${{\mathbb {M}}}(I,J)$ as follows: the vertices of $\Gamma _n$ have the vector spaces $V_i$. The maps $x_i,y_i$ are:

$$\begin{aligned} x_i:V_{i-1}\rightarrow V_i \text{ acts } \text{ as } \left\{ \begin{array}{rl} N(0) &{} \hbox { if}\ i=1 \\ M(0) &{} \hbox { if}\ i=2 \\ N(2) &{} \hbox { if}\ i=3 \\ M(2) &{} \hbox { if}\ i=4 \\ N(1) &{} \hbox { if}\ i=5 \\ M(1) &{} \hbox { if}\ i=6 \end{array}\right. y_i:V_{i}\rightarrow V_{i-1} \text{ acts } \text{ as } \left\{ \begin{array}{rl} M(0) &{} \hbox { if}\ i=1 \\ N(0) &{} \hbox { if}\ i=2 \\ M(2) &{} \hbox { if}\ i=3 \\ N(2) &{} \hbox { if}\ i=4 \\ M(1) &{} \hbox { if}\ i=5 \\ N(1) &{} \hbox { if}\ i=6. \end{array}\right. \end{aligned}$$

We can define a rank 2 module in ${\mathcal {F}}_{k,n}$ more generally. Let *I* and *J* be tightly 3-interlacing, write $I{\setminus } J$ as $\{a_1,a_2,a_3\}$ and $J{\setminus } I=\{b_1,b_2,b_3\}$, so that $a_1<b_1<a_2<b_2<a_3<b_3$. For the arrows incident with the $a_i$ and the $b_i$, we use the construction from Example 3.23 and extend it using the maps $D_1$ and $D_2$ according to whether an index belongs to both *k*-subsets or to none.

#### Definition 3.24

Let *I*, *J* be tightly 3-interlacing *k*-subsets of [*n*]. At the vertices of $\Gamma _n$, ${{\mathbb {M}}}(I,J)$ has the $V_1,\dots , V_n$. We define the maps $x_i,y_i$ as follows:

$$\begin{aligned} \begin{array}{rl} x_i:&{} V_{i-1}\rightarrow V_i \\ &{} \text{ acts } \text{ as } \end{array} \left\{ \begin{array}{rl} M(0) &{} \hbox { if}\ i=a_1 \\ N(2) &{} \hbox { if}\ i=b_1 \\ M(2) &{} \hbox { if}\ i=a_2 \\ N(1) &{} \hbox { if}\ i=b_2 \\ M(1) &{} \hbox { if}\ i=a_3 \\ N(0) &{} \hbox { if}\ i=b_3 \\ D_1 &{} \hbox { if}\ i\in I\cap J\\ D_2 &{} \hbox { if}\ i\in I^c\cap J^c \end{array}\right. \begin{array}{rl} y_i: &{}V_{i}\rightarrow V_{i-1} \\ &{} \text{ acts } \text{ as } \end{array} \left\{ \begin{array}{rl} N(0) &{} \hbox { if}\ i=a_1 \\ M(2) &{} \hbox { if}\ i=b_1 \\ N(2) &{} \hbox { if}\ i=a_2 \\ M(1) &{} \hbox { if}\ i=b_2 \\ N(1) &{} \hbox { if}\ i=a_3 \\ M(0) &{} \hbox { if}\ i=b_3 \\ D_2 &{} \hbox { if}\ i\in I\cap J\\ D_1 &{} \hbox { if}\ i\in I^c\cap J^c. \end{array}\right. \end{aligned}$$

The above construction in fact can be given a bit more generally without specifying the entries in the upper right corner of the $2\times 2$-matrices by taking the six matrices $M(c_1), N(c_2), M(c_3)$, $M(c_4), N(c_4), M(c_5), N(c_6)$ for the actions of six arrows $x_{a_1}, x_{b_1}, x_{a_2}, x_{b_2}, x_{a_3}, x_{b_3}$ and their counterparts for the *y*-arrows where the 6-tuple $(c_1,\dots , c_6)$ satisfies the conditions $t\not \mid (c_{2i+1}+c_{2i})$ for $i=0,1,2$, see [4, Section 3].

#### Exercise 3.25

Check that ${{\mathbb {M}}}(I,J)\in {\mathcal {F}}_{k,n}$. For this, check that $xy=yx$ and $x^k=y^{n-k}$ at all vertices (hence is a *B*-module) and that ${{\mathbb {M}}}(I,J)$ is free over the center.

#### Question 2

When is ${{\mathbb {M}}}(I,J)$ indecomposable? First answers are given in [3]. In particular, it is known that if a rank two module with filtration ${{\mathbb {M}}}_I/{{\mathbb {M}}}_J$ is indecomposable, then *I* and *J* have to be tightly 3-interlacing.

We show in [4] that the modules arising from Definition 3.24 are indecomposable (under the assumption that the *k*-subsets are tightly 3-interlacing). We give a definition for more general pairs of *k*-subsets and determine endomorphism rings to find indecomposability conditions and direct summands in the rank 2 module decomposes.

### Friezes from ${\mathcal {F}}_{k,n}$

In the last part, we recall how the Grassmannian cluster categories give rise to friezes, certain patterns of numbers on the Auslander–Reiten quivers of the associated category. The main reference is [5]. Let ${\mathcal {F}}_{k,n}$ be of finite type.

#### Definition 3.26

A **mesh frieze** $M_{k,n}$ **for** ${\mathcal {F}}_{k,n}$ is a collection of positive integers, one for each indecomposable of ${\mathcal {F}}_{k,n}$ (up to isomorphism), such that $M_{k,n}(P)=1$ for every indecomposable projective *P* and such that all mesh relations evaluate to 1. In order words: whenever we have an Auslander–Reiten sequence $A\rightarrow \oplus _i B_i \rightarrow C$ with $B_i$ indecomposable, $M_{k,n}(A)\cdot M_{k,n}(C)=\prod _iM_{k,n}(B_i)+1$.

#### Remark 3.27

A mesh frieze $M_{2,n}$ is a Conway–Coxeter frieze, also called $\hbox {SL}_2$-frieze. Such friezes are in bijection with triangulations of polygons [12, 13] and thus arise from specializing a cluster-tilting object in a cluster category ${\mathcal {F}}_{2,n}$ of type $\hbox {A}_{n-3}$ to 1.

#### Remark 3.28

The friezes of Dynkin types of [1] correspond to our mesh friezes for ${\underline{{\mathcal {F}}_{k,n}}}$ in types A, $\hbox {D}_4$, $\hbox {E}_6$, $\hbox {E}_8$.

#### Definition 3.29

An $\hbox {SL}_3$ **-frieze** is an array starting and ending with $k-1$ rows of 0s and with finitely many rows of positive integers in between, arranged as below, and such that each $3\times 3$ matrix has determinant 1. The **width** of an $\hbox {SL}_3$-frieze is the number of rows of positive integers between the two rows of 1s:

#### Proposition 3.30

Let $n\in \{6,7,8\}$. There is a bijection:

$$\begin{aligned} \{\hbox { mesh friezes for}\ {\mathcal {F}}_{3,n}\} {\mathop {\longleftrightarrow }\limits ^{1:1}} \{{\hbox {SL}_3\hbox {-friezes of width }n-4}\}. \end{aligned}$$

For $n=6$, this is in [28], for $n=7,8$ in [5].

By the above result, to study mesh friezes for $\mathcal F_{3,n}$, it is equivalent to study $\hbox {SL}_3$-friezes of width $n-4$. We will use the two notions interchangeably.

Most of the mesh friezes for $k=3$ arise from specializing a cluster-tilting object in ${\mathcal {F}}_{3,n}$ to 1, but there are also other mesh friezes $M_{3,n}$, see Remark 3.31. If a mesh frieze arises from specializing a cluster-tilting object to 1, it is called **unitary**. Otherwise, the mesh frieze is non-unitary.

#### Remark 3.31

The number of mesh friezes for ${\mathcal {F}}_{3,n}$ are not known for $n=8$:

$$\begin{aligned} \begin{array}{l|r|r|r} n &{} 6 &{} 7 &{} 8\\ \hline \text{ unitary } &{} 51 &{} 868 &{} 26952\ ? \\ \hline \text{ non-unitary } &{} 1 &{} 35 &{} 1872\ ? \end{array} \end{aligned}$$

The non-unitary $\hbox {SL}_3$ frieze of width 2 arises from specializing the non projective–injective summands of a cluster-tilting object to 2’s. Cuntz–Plamondon prove in an appendix to [5] that the number of non-unitary $\hbox {SL}_3$-friezes of width 3 is 35. These arise from specializing the non projective–injective summands of a cluster-tilting object to four 2s and two 1s. See Example 3.33.

#### Remark 3.32

We can use Iyama–Yoshino reduction to show that all known non-unitary mesh friezes arise from non-unitary friezes of type $\hbox {D}_4$ (i.e., from the non-unitary mesh frieze for ${\mathcal {F}}_{3,6}$ or from type $\hbox {D}_6$ (these are in [16]) or from specializing the non projective–injective summands of a cluster-tilting object of ${\mathcal {F}}_{3,8}$ to 3s. See Example 3.33.

#### Example 3.33

The mesh frieze for ${\mathcal {F}}_{3,6}$ arises from (two ways of) specializing a cluster-tilting object to 2s. The quiver of the endomorphism algebras (of the non projective–injective summands) of these two ways are below. For ${\mathcal {F}}_{3,8}$, we obtain 4 non-unitary mesh friezes by specializing (the non projective–injective summands of) a cluster-tilting object to 3s. The quiver of the endomorphism algebra is on the right. In these mesh friezes, **all** entries are $>1$:
